# Augusta Academy of Medicine

**Published:** 1895-01

**Authors:** 


### SOCIETY REPORTS.

### AUGUSTA ACADEMY OF MEDICINE.

AUGUSTA, GA., November 7, 1894.

*President Jos. Eve Allen, M.D., in the Chair.*

DR. W. Z. HOLLIDAY read a paper on "The Management of the New-born Infant." (See page 647.)

DR. ALLEN regarded the paper as one of the most interesting and valuable that had ever been presented before the Academy; he was obliged, however, to differ with the essayist in certain of his views. First, he did not regard cotton cord, or string, as a safe material to ligate the funis with; he always used for this purpose flat silk elastic cord. This material could be rendered thoroughly aseptic by soaking in an etherial solution of iodoform, and it followed down the cord as it shrank, and effectually prevented hemorrhage. Glycerine, chemically pure, is the best dressing for the stump, being far superior to vaseline or oil, because it is a bland antiseptic, and its hydroscopic properties greatly facilitate the separation of the cord. A moist is better than a dry dressing for the umbilical stump, for often the application of powders causes it to shrivel up into a hard, dry mass, which does not drop, but becomes a source of irritation and great discomfort. Such cords often require to be cut off at the base, thus subjecting the infants to the risk of hemorrhage. Bichloride gauze is not a proper material to use on the umbilicus, because it irritates the surrounding skin and produces eruption, which is very hard to cure; hence plain sterilized gauze should be used to develop the stump of the funis. The baby's belly-band should always be made of flannel. Flannel being soft and elastic, the bandage perfectly keeps its shape and protects the abdomen from cold, and thus prevents colic and congestion. He differed totally from Dr. Holliday in his objection to the wearing of flannel next the infant's skin; it should be so worn summer and winter by nursing infants, because an infant always perspires freely, and flannel absorbs the sweat, and thus makes the child more comfortable and less liable to take cold. We see the truth of this illustrated by the laborers on the streets, who always wear flannel shirts regardless of the season, stating that they are cool in summer and warm in winter. A linen or cotton shirt becomes wet, cold, and clammy when the baby is nursing or asleep, and is thus a source of great danger. Still-born infants are of two distinct and very different classes, and it is necessary to distinguish between them, because methods of resuscitation vary widely, and the one to be employed is directly dependent upon the conditions presented by the case in hand. No one method is applicable to all cases. Clinically, still-born infants are either anemic or congested. When of the first, or anemic class, the child is cold, white, and bloodless, its lips are colorless, it does not breathe, and the heart-beat can just be felt. This condition is generally caused by an interruption to the fetal circulation from premature separation of the placenta, compression of the umbilical cord, or injury to the brain from long continued pressure in the pelvic cavity, or the use of instruments. It may also be due to hydremia from malarial, syphilitic, or other blood-poisoning of the mother or infant. The prognosis is very unfavorable, as such cases seldom recover. The treatment consists in giving repeated hypodermics of brandy, and insufflating the lungs by applying the operator's mouth to the lips of the child and blowing gently and intermittently, compressing the chest when fully expanded by the hands. The infant should be suspended with its head down, so as to favor the passage of blood to the brain, and the cord should not be cut as long as there is any pulsation in it, for thus there is added four or five ounces of blood to the circulation. External means of maintaining the body heat should also be employed for hours. Efforts at resuscitation should never be given up in any case as long as the heart beats. In the congested form of still-birth, the infant is born swollen and black in the face, the eyes protrude, and a thick mucus fills up the mouth, nose, and throat. The heart's action is strong, irregular and tumultuous. This state results from a stoppage of the cerebral venous circulation, either from arrest of the shoulders after the head is born, or from the cord being tightly wound around the neck. The prognosis is favorable, for with proper care recovery is always the rule. The treatment is to cut the cord before tying, and allow it to bleed two or three ounces, remove mucus from the mouth and throat, and endeavor to excite reflex respiration by the application of whisky to the skin and dancing the child first in hot and then in cold water. Never hang the head down in such cases, as this increases the trouble by adding to the congestion of the brain.

As to the incubation of premature infants, Dr. Allen said that he had recently experimented in that line and had very remarkable results He was satisfied that many infants could thus easily be saved who otherwise would be inevitably lost. With a Credé incubator he had succeeded in rearing a six months' fetus. It was kept in the incubator three months, and came out strong and well. Premature infants, during incubation, should be disturbed as little as possible, and never washed with water, but should be rubbed with sweet or pure cod-liver oil twice daily; they should be fed at regular intervals, and the temperature of the apparatus should be kept at about 100° or 102° F. The thermometer requires constant watching, because it sometimes suddenly falls. With this six months' fetus during incubation the food used was malted milk, which was at first given in ten-drop doses repeated every five minutes for twenty or thirty minutes, and then an hour or two was allowed to elapse before another feeding. This infant always indicated when it required feeding by becoming restless, and whenever the temperature became too high or too low it would begin to whine and moan. It was kept upon its right side all the time to favor the closure of the foramen ovale. A new-born full term infant should also always be placed upon its right side, not to cause the closure of this foramen, because at this time it is generally fully occluded, but for the reason that the large liver presses upon the stomach when the child lies on the left side, and gives rise to gastric disturbances, especially after nursing.

The rearing of premature infants by incubation is best carried out in hospitals, as it is too complicated a matter for private practice or amateur nurses.

DR. THOS. D. COLEMAN said that there were only a few remarks that he desired to make on this subject. Dr. Holliday had covered the ground very fully, but had left out, doubtless unintentionally, a very important point. In the new-born failure to respire is, so to speak, mechanical in many instances. That is to say, the larynx and glottis may be occluded by tenacious mucus, so that, though the breathing movements may be attempted, they cannot be carried out because the air cannot gain entrance to the lungs. When this is the case, it may be removed either with the finger or with a catheter run down to the level of the epiglottis, by which means the mucus may be sucked out.

He wished to comment upon Dr. Holliday's method of artificial respiration. It is not in any way superior or equal to the Sylvester method, and for these reasons: asphyxia even in adults has its different stages, and a point is reached when all the reflexes are abolished; with this abolition there is muscular relaxation, which affects the tongue and muscles of the pharynx as well as the skeletal muscles. The tongue here tends to drop back into the pharynx and cover the glottis, so that such respiratory movements of the chest that were developed by the method suggested by Dr. Holliday would be neutralized by the presence of the tongue over the glottis. It would seem then that a method such as Sylvester has suggested in which the tongue is held forward while the respiratory movements of the chest are carried on artificially, is highly desirable.

With regard to the cutting of the cord, Dr. Coleman wishes to emphasize a point that has not been touched upon. No physician is justified in needlessly exposing his patients, but in this stage of the delivery it is needful for the physician to see what he is doing. The practice of ligaturing and cutting the cord, therefore, beneath the cover, depending entirely upon the sense of touch, should be condemned, for one authority, at least, notes the fact that more than one hapless male infant has lost his penis by this procedure.

The doctor's comment on the pins for the binder was a good one; the style of pin he suggests is all right, but be careful to include only the binder. The mother of a now healthy adult had told Dr. Coleman no longer than a month ago that, when an infant in his puny days, her son was treated nearly seventy-two hours for the colic before it was found out that the nurse had included a thickness of skin in one of the pins that held the binders.

DR. A. S. TINSLEY said that when it is considered that about fifty per cent. of the blindness in many places is caused by ophthalmia neonatorum due to infection at birth, then the importance of prophylaxis may be more correctly appreciated.

The result of systematic prophylactic measures in some of the large lying-in hospitals has been most gratifying, and has succeeded in almost entirely banishing the disease from the wards of the well regulated hospitals.

In every case where there is present any vaginal discharge, a solution of corrosive sublimate 1 to 2000 should be used during labor. As soon as the eyes are thoroughly cleansed after birth, three drops of a solution of nitrate of silver, ten grains to one ounce of distilled water, should be dropped into the conjunctival sac.

DR. HOLLIDAY, in conclusion, said that Dr. Allen's idea of using the elastic ligature, previously rendered aseptic, is in no sense objectionable. His own idea was, however, that more depends on the way it is applied than on the ligature itself. He did not think that there was any absorption from the cortex of the cord, and after using a great many things for the purpose, considers the cotton cord, which is hardly twisted at all, the equal of any. It is broad enough not to cut the tissues, does not easily slip, and in his hands had been highly satisfactory. The bichloride gauze, after being covered with vaseline on the under surface, will not do harm, but the plain sterilized answers equally well. In making his statement as to the use of flannels at the first dressing of baby, he emphasized the qualifying words *coarse* and *hot weather*, intending that it should be applied more particularly to those cases where the fine soft fabric which the wealthy furnish cannot be afforded. In the very hot weather of our mild summer, he had frequently wrapped the body in light blankets after dressing, and they can easily be kept warm enough in this way.

DR. LAMB'S suggestion that the eyes of the baby be wiped off with a clean rag before removing from bed in certain cases is an excellent one, and he was obliged to him for it. Dr. Lamb's observation, too, that some babies are restless and cry on account of vesical calculi is sustained by his experience. He has never cut the cord very close to the abdomen, but sees no reason why it should not do well.

DR. COLEMAN'S statement concerning the removal of mucus from the throat is an amplification of what is implied by artificial respiration. If the child is inverted as he described, gravity alone causes the mucus to run out. His reference to the use of safety pins was an instance of carelessness in the application of it.

---

### TWO EASY AND DELICATE TESTS FOR ALBUMIN IN URINE.

Dr. C. Fouchlos (*Progres Medical*) recommends two new tests for albumin in urine, for which he claims utmost delicacy and absence of any possible fallacy.

1. Add to the suspected urine a few drops of a one per cent. solution of corrosive sublimate; in case of turbidity, add some drops of acetic acid. If the turbidity persists it is due to the presence of albumin.

2. Take 100 cc. of a ten per cent. solution of sulpho-cyanide of potassium, and mix with it 20 cc. of acetic acid. Add a few drops of this mixture to the urine. If albumin is present in small quantities, an immediate turbidity will ensue; if in larger quantities, a heavy white precipitate will appear.—*Medical Review.*

---

### ACUTE TONSILLITIS.

Salicilate of soda is recommended as little less than a specific in acute tonsillitis. It should be given as early in the attack as possible, and in sufficient doses to cause ringing in the ears. Fifteen grains every three hours will usually cause this effect, when the dose may be diminished to ten, and then to five grains at the same intervals. It should be continued a day or two after disappearance of the fever.—*N. C. Med. Jour.*

